# A_2B_ Adenosine Receptor Enhances Chemoresistance of Glioblastoma Stem-Like Cells under Hypoxia: New Insights into MRP3 Transporter Function

**DOI:** 10.3390/ijms23169022

**Published:** 2022-08-12

**Authors:** José-Dellis Rocha, Daniel Uribe, Javiera Delgado, Ignacio Niechi, Sebastián Alarcón, José Ignacio Erices, Rómulo Melo, Rodrigo Fernández-Gajardo, Flavio Salazar-Onfray, Rody San Martín, Claudia Quezada Monrás

**Affiliations:** 1 Instituto de Bioquímica y Microbiología, Facultad de Ciencias, Universidad Austral de Chile, Valdivia 5110566, Chile; 2 Millennium Institute on Immunology and Immunotherapy, Universidad Austral de Chile, Valdivia 5090000, Chile; 3 Servicio de Neurocirugía, Instituto de Neurocirugía Dr. Asenjo, Santiago 7500691, Chile; 4 Instituto de Ciencias Biomédicas, Facultad de Medicina, Universidad de Chile, Santiago 7500691, Chile; 5 Millennium Institute on Immunology and Immunotherapy, Facultad de Medicina, Universidad de Chile, Santiago 7500691, Chile

**Keywords:** MRP3, adenosine, A_2B_, glioblastoma, chemoresistance, GSCs, hypoxia

## Abstract

Glioblastoma is the most common and aggressive primary brain tumor, characterized by its high chemoresistance and the presence of a cell subpopulation that persists under hypoxic niches, called glioblastoma stem-like cells (GSCs). The chemoresistance of GSCs is mediated in part by adenosine signaling and ABC transporters, which extrude drugs outside the cell, such as the multidrug resistance-associated proteins (MRPs) subfamily. Adenosine promotes MRP1-dependent chemoresistance under normoxia. However, adenosine/MRPs-dependent chemoresistance under hypoxia has not been studied until now. Transcript and protein levels were determined by RT-qPCR and Western blot, respectively. MRP extrusion capacity was determined by intracellular 5 (6)-Carboxyfluorescein diacetate (CFDA) accumulation. Cell viability was measured by MTS assays. Cell cycle and apoptosis were determined by flow cytometry. Here, we show for the first time that MRP3 expression is induced under hypoxia through the A2B adenosine receptor. Hypoxia enhances MRP-dependent extrusion capacity and the chemoresistance of GSCs. Meanwhile, MRP3 knockdown decreases GSC viability under hypoxia. Downregulation of the A_2B_ receptor decreases MRP3 expression and chemosensibilizes GSCs treated with teniposide under hypoxia. These data suggest that hypoxia-dependent activation of A_2B_ adenosine receptor promotes survival of GSCs through MRP3 induction.

## 1. Introduction

Glioblastoma (GBM) is the most common and aggressive primary brain tumor [1,2,3], characterized by elevated cell proliferation, robust angiogenesis, and extensive hypoxic areas associated with necrosis [4]. Despite multimodal treatments, which include surgical resection followed by radiotherapy and chemotherapy, the median survival time does not exceed 15 months [5]. It has been suggested that therapeutic failure in GBM can be attributed to a cellular subpopulation with stem properties called glioblastoma stem-like cells (GSCs) [6,7,8,9]. These cells have enhanced chemoresistance potential compared to other GBM cells [8,10] and have been proposed as the main factor responsible for tumor recurrence after treatments [6,7,8,11]. Malignant properties of GSCs can be promoted by the tumor’s microenvironmental conditions—such as hypoxia—where these cells persist [12,13,14]. It has been reported that hypoxia regulates several aspects of GSC biology through HIFs such as stemness, angiogenesis, and cell invasion/migration [15,16,17,18,19,20]. Likewise, hypoxia is related to the promotion of chemoresistant phenotypes of GSCs [21,22]. However, the mechanisms by which hypoxia promotes drug resistance in these cells have been poorly studied. One of the most important mechanisms related to chemoresistance in cancer is the expression and activity of ATP-binding cassette (ABC) family transporters [23,24]. These transporters extrude drugs to the extracellular space, protecting cells against cytotoxic effects [25]. The expression of ABC transporter family members such as MDR-1, ABCG2, MRP1, and MRP3 has been documented in several cancers, including GBM. Additionally, its elevated levels have been clinically correlated with poor prognosis [26,27,28,29,30,31,32]. Moreover, MRP3 expression is higher than MDR1 and ABCG2 in glioma models, but this transporter has not been studied as extensively as others [32,33,34,35,36]. For example, etoposide and doxorubicin are substrates of MRP1 and MRP3, but teniposide is specific to MRP3, and not MRP1 [37]. MRP3 is involved in the chemoresistance of breast cancer [38], hepatocellular carcinoma [39], and colon cancer [40], among other types. In GBM, elevated MRP3 transcript levels are correlated with high death risk, and it was proposed as a new immunotherapy target [27]. However, the regulation of MRP3 expression is not well understood. Adenosine is a nucleoside with several functions in the central nervous system, such as modulating neurotransmitter release, inducing synaptic plasticity and acting as a neuroprotector against ischemia and hypoxia [41,42]. However, high levels of adenosine have also been reported in the hypoxic tumor microenvironment, suppressing the immune response and promoting neovascularization and tumor growth [43,44]. Adenosine signaling is mediated by P1 family receptors, including A1, A2A, A2B, and A3 [45]. These receptors are coupled to different G proteins and have different ligand affinity; A2B and A3 are classified as low-affinity adenosine receptors [45]. Extracellular adenosine levels are increased in GSCs in relation to differentiated cells. Its signaling through A3 promotes MRP1 expression, thereby enhancing chemoresistance under normoxia [46]. We and others have reported that, under hypoxic conditions, the production and accumulation of extracellular adenosine is aberrantly increased in GSCs [19,20,47]. However, its role in MRP-dependent chemoresistance under hypoxia is unknown. The A2B receptor has the lowest affinity and its expression is regulated by HIF-1α under hypoxia [48]. The A2B receptor is expressed in the hypoxic microenvironments of several solid tumors, including colon [49], prostate [50], lung [51,52], and breast cancer [53]. It has also been associated with proliferation, tumor growth, and metastasis [54]. However, the role of the A2B adenosine receptor on GSC chemoresistance under hypoxia is unknown. Here, we evaluate the chemoresistance phenotype of hypoxic GSCs, highlighting the roles of the A2B adenosine receptor and MRP3 transporter.

## 2. Results

### 2.1. MRP3 Induction in GSCs under Hypoxia

**Figure 1:**
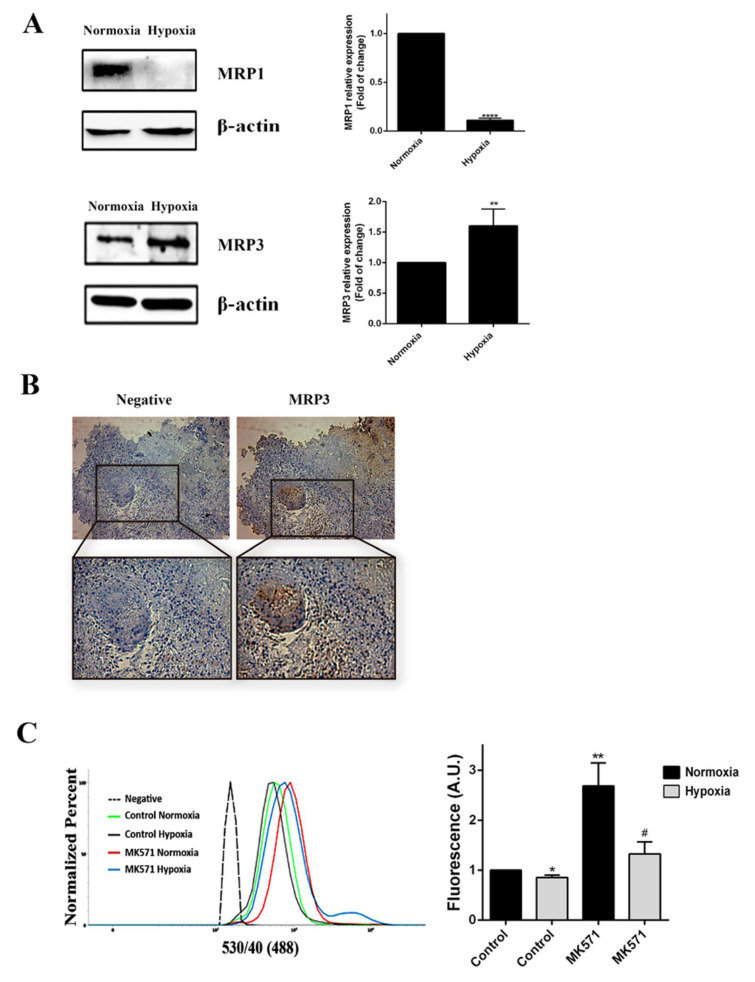
MRP3 expression in GSCs under hypoxic conditions. (**A**) MRP3 protein levels from GSCs under normoxia and hypoxia were analyzed by Western blot of GSCs. Bands were quantified and plotted. β-actin was used as a housekeeping control. (**B**) MRP3 expression was detected in human GBM samples by immunohistochemistry (scale bar 50 m). (**C**) Histograms represent fluorescence of CFDA accumulation (left) and plotted as fold of change of fluorescence (right). *n* = 3, * *p* < 0.05, ** *p* < 0.01, **** *p* < 0.0001, # *p* < 0.05 relative to hypoxia control.

We assessed whether MRP3 expression was mediated by oxygen tension. We found that MRP3 expression was induced under hypoxia (Figure 1A), suggesting that this transporter plays a role in hypoxic GSCs. Additionally, through immunohistochemical analysis, we detected MRP3 expression in human GBM samples, especially in discrete pseudopalisade areas, which is a histopathological characteristic associated with the hypoxic/necrotic niche (Figure 1B). To determine the MRP-dependent extrusion capacity in GSCs, we performed a CFDA 193 accumulation assay at different oxygen tension levels. We observed that intracellular CFDA fluorescence is 20% lower under hypoxia compared to normoxia (Figure 1C), suggesting a higher extrusion capacity of hypoxic GSCs. Treatment with the MRP inhibitor, 196 MK571, increases the fluorescence associated with CFDA accumulation in both normoxic and hypoxic GSCs, indicating the presence of active MRP transporters (Figure 1C). These results suggest that MRP3 could be involved in the extrusion capacity and chemoresistance 199 of GSCs under hypoxia.

### 2.2. Hypoxia Enhances Chemoresistance to Doxorrubicin and Teniposide

**Figure 2:**
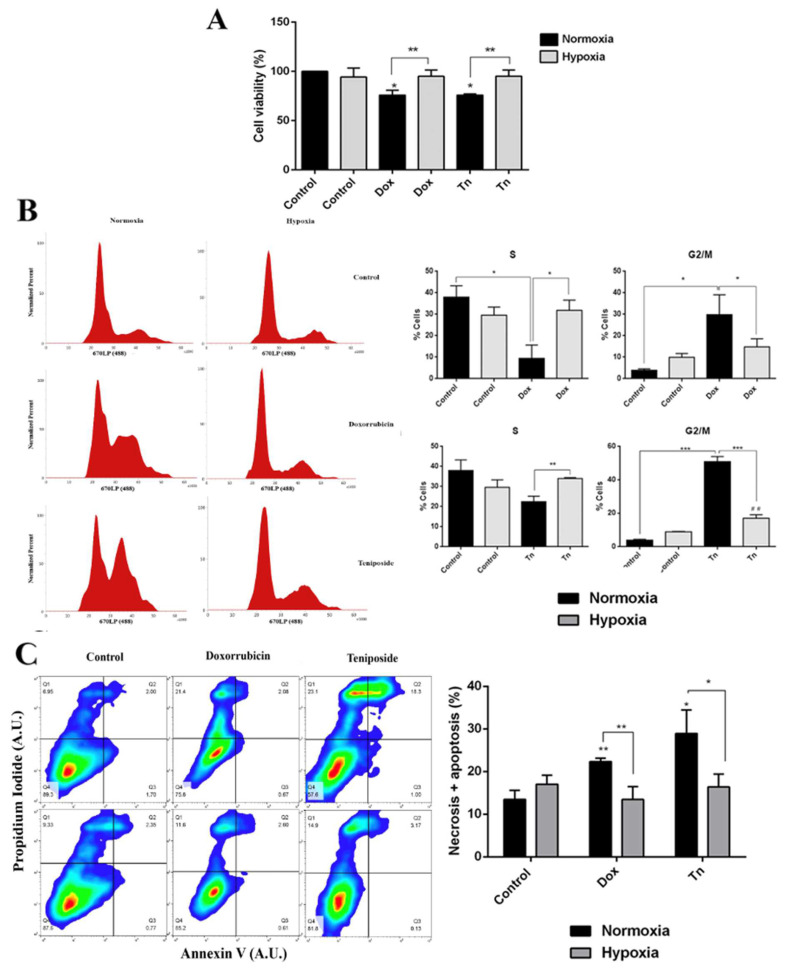
Cytotoxic effect of doxorubicin and teniposide on GSCs under normoxia and hypoxia. (**A**) GSC viability was measured by MTS in GSCs treated with 0.5 µM doxorubicin (Dox) or 0.5 µM teniposide (Tn) under normoxia or hypoxia. (**B**) Histograms representing the cell cycle profile data from the S phase (left) and the G2/M phase were plotted (right). (**C**) Apoptosis assay. Representative dot plot obtained from 10,000 cells (left) and data from necrotic and apoptotic cells were plotted (right). *n* = 3, * *p* < 0.05, ** *p* < 0.01, *** *p* < 0.001, ## *p* < 0.01 relative to hypoxia control.

To determine whether higher MRP3 protein levels in hypoxic GSCs are associated with enhanced chemoresistance, we conducted viability, cell cycle, and apoptosis assays with the MRP3 substrates, doxorubicin and teniposide (Figure 2). As expected, both treatments decreased cell viability under normoxia, while no changes were observed under hypoxia (Figure 2A). Cell cycle assays demonstrated that the cell population in the G2/M phase increased when the S phase population decreased under drug treatments, but this effect was partially prevented under hypoxia (Figure 2B). As expected, the percentage of necrotic and apoptotic cells increased in drug treatments under normoxia, but no changes were observed under hypoxia (Figure 2C). Together, these results suggest that hypoxia promotes resistance to the MRP3 substrates, doxorubicin and teniposide.

### 2.3. Knockdown of MRP3 Differentially Impairs GSC Viability under Hypoxia

**Figure 3:**
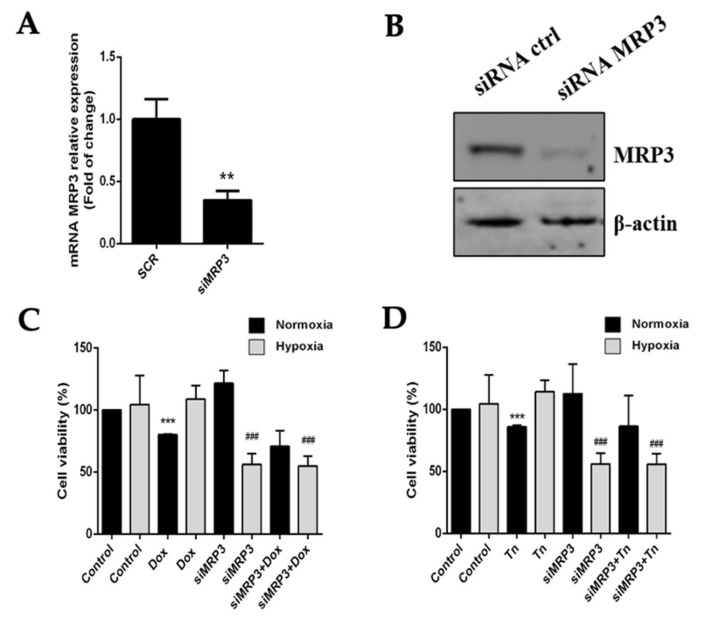
Effect of MRP3 silencing in GSCs under normoxia and hypoxia. (**A**) MRP3 knockdown was confirmed by RT-qPCR (**A**) and Western blot (**B**). Viability of GSCs-MRP3^KD^ was measured by MTS assay after a 24 h treatment with 0.5 µM doxorubicin (Dox) (**C**) or 0.5 µM teniposide (Tn) (**D**). *n* = 3, ** *p* < 0.01, *** *p* < 0.001, ### *p* < 0.001 relative to hypoxia control.

To evaluate the role of MRP3 in the viability of hypoxic GSCs, we used a specific siRNA to silence its expression. MRP3 knockdown (KD) was confirmed by RT-qPCR and Western blot (Figure 3A,B). GSCs-MRP3KD were treated with doxorubicin or teniposide under normoxia and hypoxia, demonstrating that MRP3 knockdown decreased GSC viability under hypoxia, but no changes were observed under normoxia (Figure 3C,D). This suggests a role of MRP3-dependent viability under hypoxia. No additive or synergistic effects were observed in GSCs-MRP3KD treated with doxorubicin (Figure 3C) or teniposide (Figure 3D). These results suggest that MRP3 expression plays a critical role in GSC viability exclusively under hypoxic conditions.

### 2.4. MRP3 Over-Expression under Hypoxia Is Regulated by A_2B_ Adenosine Receptor

**Figure 4:**
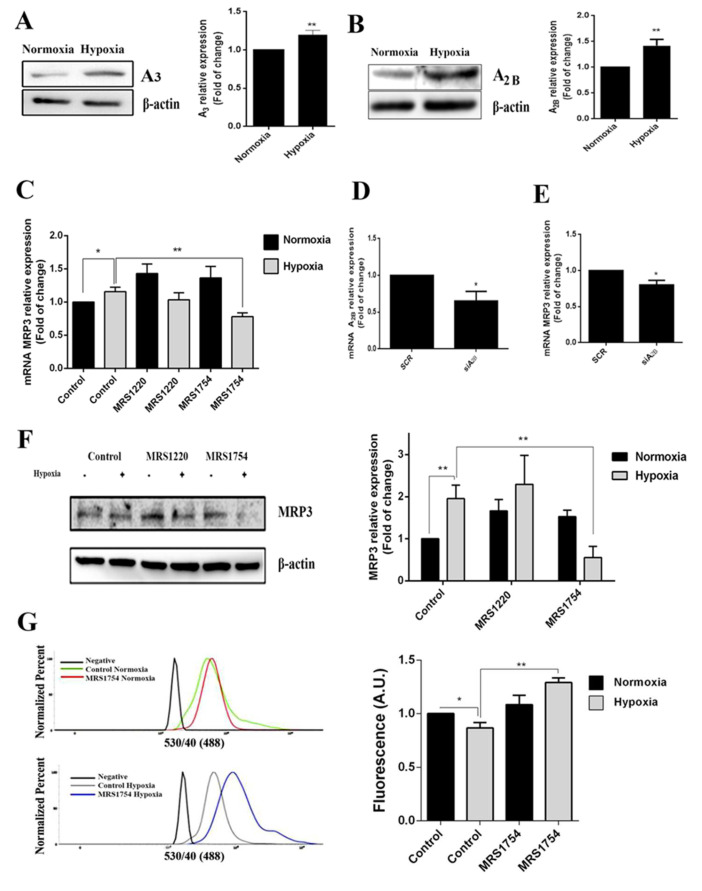
Role of adenosine in MRP3 expression in GSCs. GSCs were treated with 10 uM MRS1220 or 50 nM MRS1754. (**A**) A_3_ adenosine receptor protein levels in GSCs under normoxia and hypoxia were analyzed by Western blot. The bands were quantified and plotted. (**B**) As described in A, except for the A_2B_ adenosine receptor. (**C**) MRP3 mRNA levels were analyzed by RT-qPCR. (**D**) A_2B_ knockdown was confirmed by RT-qPCR. (**E**) MRP3 mRNA levels were analyzed by RT-qPCR in A_2B_ knockout hypoxic GSCs. (**F**) MRP3 protein levels in GSCs were analyzed by Western blot. (**G**) Extrusion capacity was analyzed by flow cytometry using GSCs. Histograms represent the fluorescence of CFDA accumulation (left) and are plotted as a fold of change in fluorescence (right). *n* = 3, * *p* < 0.05, ** *p* < 0.01.

Adenosine accumulation was aberrantly increased in GSCs under hypoxia; therefore, we determined whether its signaling was involved in hypoxia-dependent MRP3 over-expression. Low-affinity A3 and A2B adenosine receptor expression increased in hypoxic GSCs (Figure 4A,B) and A2B blockade with MRS1754 decreased MRP3 transcript and protein levels under hypoxia. However, this effect was not observed with MRS1220 (A3 antagonist) nor in normoxic conditions (Figure 4C,F). Additionally, A2B knockdown (Figure 4D and Figure S1) decreased MRP3 transcript levels in hypoxic GSCs (Figure 4E), suggesting that MRP3 induction under hypoxia is mediated by the A2B adenosine receptor. To determine the effect of the A2B blockade on GSC extrusion capacity, we performed CFDA accumulation assays. The A2B blockade with MRS1754 increased the CFDA accumulation in hypoxic GSCs, while no changes were observed in normoxic conditions (Figure 4G). Together, these results suggest that MRP3 over-expression and MRP activity induction under hypoxia are mediated by the A2B adenosine receptor.

### 2.5. Blockage of A_2B_ Adenosine Receptor Chemosensitizes Hypoxic GSCs to Teniposide

**Figure 5:**
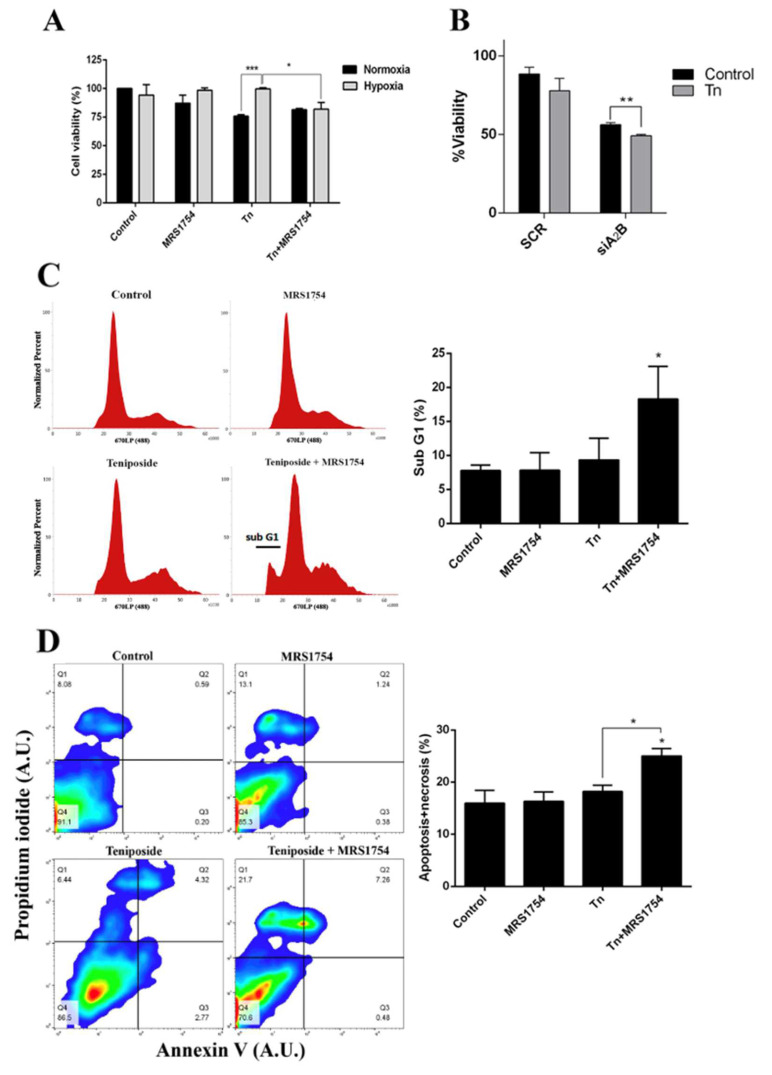
Effect of blockage of A_2B_ in combination with the MRP3 drug substrate on GSCs under hypoxia. (**A**) GSC viability was measured by MTS assays of cells under both normoxia or hypoxia treated with 0.5 µM teniposide (Tn) alone or in combination with 50 nM MRS1754 for 24 h. (**B**) GSC viability was measured by MTS assays with GSC knockdown for A2B under hypoxia treated with 0.5 µM teniposide (Tn) for 24 h. (**C**) Cell cycle analysis was performed with hypoxic GSCs treated with 0.5 µM teniposide (Tn) alone or in combination with 50 nM MRS1754 for 24 h. The histogram represents the cell cycle profile (left) and quantification (right). (**D**) The apoptosis assay was performed with hypoxic GSCs treated as in C. The representative dot plot obtained from 10,000 cells (left) and data from necrotic and apoptotic cells were plotted in columns (right). *n* = 3, * *p* < 0.05, ** *p* < 0.01, *** *p* < 0.001.

Since the blockage of A_2B_ adenosine receptor prevents MRP3 induction and the extrusion capacity of GSCs under hypoxia, we evaluated the possible effects on hypoxia-dependent chemoresistance to an MRP3 substrate, teniposide (Figure 5). As expected, teniposide alone decreased GSC viability under normoxia, but not under hypoxia (Figure 5A). This confirms enhanced chemoresistance under hypoxia. Despite this, A_2B_ blockage with MRS1754 or A_2B_ knockdown under hypoxia in combination with teniposide decreased GSC viability (Figure 5A,B). Finally, flow cytometry analysis showed that sub-G1 populations (Figure 5C) and the apoptosis/necrosis population (Figure 5D) do not change with either teniposide or MRS1754 treatments; it exclusively increased under combined treatment. These results suggest that A_2B_ enhances teniposide chemoresistance of GSCs under hypoxia and the use of teniposide in combination with A_2B_ blockage could be a new strategy to target hypoxic GSCs.

## 3. Discussion

The expression and activity of ABC transporters, especially MRPs, has been a challenge in recent years due to its structural and functional versatility. It has emerged as a strategy to target GSC’s chemoresistance. Although perivascular and hypoxic niches have been described for GSC persistence, low oxygen pressures are strongly related to cells’ aggressiveness by promoting the maintenance of stem properties and drug resistance. However, GSC chemoresistance mechanisms induced under hypoxia have not been well characterized. Here, we have identified for the first time the hypoxia-dependent MRP3 induction in GSCs through the A2B adenosine receptor as well as its role on cell viability and chemoresistance.

### 3.1. MRP3 as a Compensatory Transporter

Although MRP1 promotes GSC chemoresistance under standard culture conditions [46], we demonstrated that MRP3 expression is induced under hypoxia. Moreover, MRP3 was detected in human GBM samples, with a strong signal in pseudo-palisades, which is associated with hypoxic/necrotic niches within the tumor [55]. MRP3 expression was described in GBM tissue and cell lines, and its function has been related to chemoresistance and the risk of a poor prognosis [27,34,56,57,58]. These and our results suggest MRP3 as a tumor marker and a potential target for GBM [27,59]. Although GSCs express MRP1, MRP3 is expressed in cells with stem-like characteristics, an effect that is favored by low oxygenation levels [60,61]. In the U251 GBM cell line, MRP1 is downregulated under temozolomide treatment and Livin knockdown (apoptosis inhibitor protein). We also found that MRP3 expression is induced [62]. Even though MRP3 is the transporter with the highest sequence identity to MRP1 [63], it is unclear why its expression is differentially regulated in response to the microenvironment. We suggest that MRP3 could be an alternative xenobiotic extrusion pathway where known MRP1 function is insufficient. However, future therapies targeting MRPs depend on understanding its function, activity, and compensatory mechanisms. Nevertheless, MRP3 has not been studied as extensively as P-gp, BCRP, or MRP1. However, this does not mean that this transporter does not have a key role in chemoresistance mediated by compensatory processes, as we proposed.

### 3.2. MRP3 Regulation: Importance of Tumor Microenvironment

The Wnt/β-catenin pathway, which regulates stemness maintenance in several cancer models, downregulates MRP3 expression in colon cancer cells [21,64,65]. MRP1 or MRP3 knockdown decreases stemness markers’ expression in breast cancer stem-like cells. However, only MRP3 knockdown decreased the population of cells with a stem phenotype and reduced tumor growth in a murine model [38], suggesting that MRP3 function is not limited only to chemoresistance. In this work, we observed that MRP3 knockdown has no effect on GSC viability or chemoresistance under normoxia. However, GSC viability decreased under hypoxia, suggesting that this transporter is crucial for GSC maintenance at low oxygen tensions with elevated oxidative damage. MRP extrusion capacity was measured with CFDA as the substrate and MK571 as an MRP inhibitor. MK571 impairs conformational changes of MRPs that are involved in its activity. However, it has been reported that this drug is highly cytotoxic for GSCs in mid- to long-term experiments. In fact, only 30 min of treatment with MK571 prior to CFDA incubation is needed to impair MRP activity. However, at this time, changes in MRP protein levels are undetected. It has been reported that MRP3 regulates cellular levels of reactive oxygen species (ROS) by extrusion into the extracellular space [40]. Thus, MRP3 could be part of a cellular mechanism to reduce oxidative damage in hypoxic GSCs by extruding ROS. Additionally, it is reported that normal stem cells and cancer stem cells maintain low baseline levels of ROS, which is related to stemness maintenance [37,66]. However, the mechanisms involved in hypoxia-dependent MRP3 function in GSCs are not fully understood.

### 3.3. Adenosine and A_2B_-Dependent MRP3 Regulation

**Figure 6:**
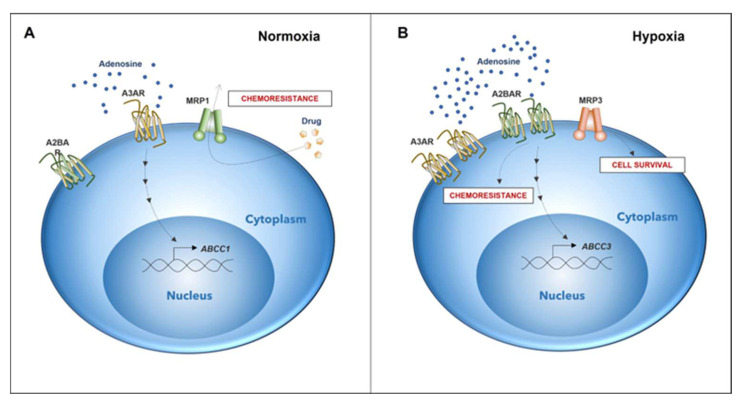
Differential expression of genes associated with chemoresistance in GSCs depends on microenvironmental oxygen levels. (**A**) Chemoresistance in GSCs under normoxic conditions is elicited by the expression of the A_3_ adenosine receptor and MRP1 transporter (DOI: https://doi.org/10.3390/10.18632/oncotarget.12033, accessed on 26 May 2022). (**B**) In GSCs under a hypoxic microenvironment, A_2B_ adenosine receptor expression increases. Activation of A_2B_ triggers the expression of MRP3. Expression and activation of A_2B_ potentially play a role in a novel mechanism of chemoresistance together with MRP3, which could be involved in GSC survival under hypoxia.

We and others have reported that hypoxic GSCs produce elevated levels of adenosine signaling through A_2B_ and/or A_3_ receptors, thereby promoting cell invasion, migration, proliferation, and chemoresistance [19,20,21,46,47]. Under normoxic conditions, A_3_ blockage or silencing decreases MRP1 expression/activity, which promotes vincristine chemosensitization of GSCs [46]. Even though A_3_ and A_2B_ are induced under hypoxia, MRP3 levels are only downregulated by A_2B_ blockage, while we observed no effects on MRP3 expression by A_3_ blockage. A_2B_ promotes the malignant phenotype of GSCs through Akt and Erk [47], suggesting two possible pathways of A_2B_-dependent MRP3 expression, considering that favorable outcomes of post-operative therapy are reported with teniposide, we aimed to explore novel mechanisms involving this drug. Here, we demonstrated that teniposide decreased A_2B_ levels under hypoxic conditions (Figure S2), suggesting that this MRP3 substrate is suitable for A_2B_ blockage co-treatment. Our results suggest that the A_2B_/MRP3 axis is involved in the intrinsically resistant phenotype of GSCs under hypoxia (Figure 6). Lack of an apparent correlation between Figure 3C and Figure 5B can be associated with A_2B_ activation. Indeed, changes in viability and synergic results shown in Figure 5B suggest a role of A_2B_ blockage in cell viability independent of drug administration and transporter regulation. Our results showed no difference between the drug alone and siMRP3 with the drug; therefore, we aimed to explain the critical role that MRP3 may play in GSCs under hypoxia beyond chemoresistance.

## 4. Materials and Methods

### 4.1. Pharmacological Agents

MRS1220 (Tocris, Bristol, UK) was used as a selective antagonist of A3AR. MRS1754 (Tocris) was used as a selective antagonist of A2BAR. Doxorubicin (Laboratorio de Chile, Santiago, Chile) and teniposide (Tocris) were used as drug substrates of MRP3.

### 4.2. Cell Line

U87MG human GBM cell line was acquired from the American Type Culture Collection Company (ATCC^®^ HTB-14TM). Cells were grown in DMEM-F12 medium supplemented with 10% fetal bovine serum, 100 U/mL penicillin, and 100 µg/mL streptomycin. All of these materials were purchased from Life Technologies (Manassas, VA, USA). Cells were incubated in standard conditions at 37 °C in a humidified atmosphere with 5% CO_2_. To generate GSCs, cells were grown in Neurobasal medium (Gibco, Waltham, MA, USA) supplemented with EGF (20 ng/mL), bFGF (20 ng/mL), 1× B27 (*w*/*o* vitamin A), and 100 units/mL of penicillin/streptomycin. For hypoxia (0.5% O_2_) experiments, a gas mixing chamber (5% CO_2_ and 95% N2 mixture) was used for GSC conditioning 24 h before pharmacologic treatments. Normoxia (21% O_2_) experiments were performed using standard culture conditions.

### 4.3. Viability Assays

MTS (Promega, Madison, WI, USA) assays were performed with 10,000 GSCs in a volume of 50 μL with Neurobasal medium and were conditioned for 24 h in normoxia or hypoxia in a 96-well plate. Cells were treated with MRS1220 (10 μM), MRS1754 (50 nM), doxorubicin (0.5 μM), and teniposide (0.5 μM) for 24 h. After treatment, 20 μL/well of MTS reagent was added and incubated for 2 h at 37 °C. Absorbance was measured at 490 nm using a microplate reader (Synergy HT, BioTek Instruments, Inc., Winooski, VT, USA).

### 4.4. Western Blot

Protein extracts (50 μg) of GSCs were separated by 8% SDS-PAGE, followed by transfer to a 0.22 μm PVDF membrane (Bio-Rad, Hercules, CA, USA) and blocked with 5% BSA. Finally, membranes were incubated with primary antibodies: anti-MRP3 (NOVUS, Centennial, CO, USA), anti-A2B (Abcam, Cambridge, UK), and anti-A3 (Santa Cruz, Dallas, TX, USA) for 16 h at 4 °C or anti-β-actin HRP-coupled (Santa Cruz) for 1 h at room temperature. Later, a secondary HRP-coupled antibody purchased from Jackson laboratories was incubated for 1 h at room temperature, then detected using SuperSignalTM West Dura (Thermo Fisher Scientific Inc., Waltham, MA, USA) by system syngene G:BOX (Synoptics Ltd., Cambridge, UK). Results were quantified in ImageJ and expressed as fold of change using β-actin as housekeeping.

### 4.5. Cell Cycle Analyses

GSCs were fixed in 70% ethanol overnight at 4 °C, then stained with 50 µg/mL propidium iodide (PI) solution (Thermo Fisher, Waltham, MA, USA) and 10 µg/mL RNAse A (Invitrogen, Carlsbad, CA, USA). Stained cells were analyzed on FACSJazz. Histograms were constructed from 10,000 events. Flow cytometric analysis was performed using FlowJo software.

### 4.6. Apoptosis Assay

A total of 400,000 GSCs were used for each experimental condition. Cells were harvested and centrifuged at 600× *g* for 5 min and supernatant was discarded, and the cells were resuspended in 500 µL of annexin-V binding buffer. Subsequently, 5 µL of annexin-V (A-V)-FITC (Abcam) and propidium iodide (PI) (50 µg/mL) was added to each sample. Samples were incubated at room temperature for 5 min for subsequent reading by flow cytometry. For dot plots construction, signal from Annexin-V-FITC was established as the X-axis. The fluorescence of propidium iodide was established as the Y-axis. The quadrants (A-V^−^, PI^−^), (A-V^+^, PI^−^), (A-V^+^, PI^+^), and (A-V^−^, PI^+^) correspond to viable cells, early apoptosis, late apoptosis, and necrosis, respectively. Data were quantified using FlowJo software from 10,000 events.

### 4.7. Extrusion Assay

Another paper describes the MRP CFDA substrate accumulation assay method [20]. Briefly, we seeded GSCs (200,000) in a 12-well plate and incubated them for 24 h under normoxic and hypoxic conditions. Drug treatments were applied for 24 h, maintaining the same conditions. MK571 (20 µM) was used as an MRP inhibitor control. After treatments, we applied 500 nM CFDA per well and incubated the plate for 30 min at 37 °C and 5% CO_2_. We analyzed the cells using FACS. Additionally, fluorescence profiles were expressed in histogram plots and the data were quantified using FlowJo software.

### 4.8. siRNA Transfection

siRNA transfection has also been described in another paper [19]. Briefly, GSCs (400,000) were seeded in Opti-MEM and incubated with a mix of 1 µg of siRNA (all purchased from Santa Cruz, Waltham, MA, USA) and 10 µL Lipofectamine 2000 (Invitrogen). After 6 h, cells were incubated with the Neurobasal medium and assays started 24 h post-transfection.

### 4.9. RT-qPCR

RNA extraction, cDNA amplification, and qPCR were described in another paper as well [20]. Briefly, qPCR was performed with Brilliant II SYBR^®^Green (Agilent Technologies, Santa Clara, CA, USA) master mix according to the manufacturer’s instructions. Primers used were as follows: MRP3 F: TGCCCCAGTTAATCAGCAACCT R: TCCTCTTGGCTCAGGAATTGCT, A2B F: GCTTCTGCACTGACTTCTACG R: TCCCCGTGACCAAACTTTTAT. ROX fluorophore was used as a reference. Additionally, relative quantification was performed using the 2^ΔΔCt^ method using β-actin as housekeeping.

### 4.10. Histological Analysis

Surgical resection procedures of GBM patients obtained tumor samples at the Departamento de Neurocirugía, Instituto de Neurocirugía Asenjo, Santiago, Chile. All procedures were carried out with the approval of the Bioethics Committee of the Universidad Austral de Chile (Permit Number: 29–2011) and Servicio de Salud Metropolitano Oriente. Human tissues were fixed in 3.7% paraformaldehyde (Sigma Aldrich, Darmstadt, Germany) and embedded in paraffin. Then, 5 μm sections were mounted on silanized slides and immunodetections were performed with primary anti-MRP3 (NBP1-42347) from NOVUS and anti-A2BAR (ab40002) from ABCAM in blocking solution overnight at 4 °C. Immunosignals were visualized using the ImmPRESS™ Excel Amplified HRP (peroxidase) Polymer Staining Kit (Vector Laboratories, Newark, CA, USA). Counterstaining was performed using hematoxylin and eosin classic stain.

### 4.11. Statistics

The results were plotted, processed, and presented according to mean ± standard deviation (S.D.) using the GraphPad Prism 6.01 program. We evaluated the statistical significance of treatments using ANOVA and the student’s t-test. A *p*-value less than 0.05 was considered significant.

## 5. Conclusions

Our results showed no difference in the treatment of MRP3 KD cells exposed to drugs. However, the decreased viability and reduced MRP3 expression in GSCs under hypoxia suggests that this protein has a critical role in the MRP3 KD cells chemoresistance under hypoxia. It is possible that MRP3 is capable of transporting biomolecules that are essential for maintaining the mitogenic characteristics of GSCs [67]. This has been demonstrated in other cell models, in which MRP3 was described as being capable of transporting lysophosphatidylinositol (LPI) in colon carcinoma cells and activating the GPCR signaling cascade; this leads to increased proliferation, clonal expansion, and, consequently, progression of pathology [67]. However, more studies are necessary to verify the existence of differential regulatory pathways involving MRP3 in our model.

## Data Availability

Details of all experiments, including data and material used for performing this study, will be made accessible. Data are either included in the manuscript or available upon request.

## Supplementary Materials

The following are available online at https://www.mdpi.com/article/10.3390/ijms23169022/s1.
