# Supplementary material for: A2B Adenosine Receptor Enhances Chemoresistance of Glioblastoma Stem-Like Cells under Hypoxia: New Insights into MRP3 Transporter Function
Source: Int J Mol Sci. 2022 Aug 12;23(16):9022. doi: 10.3390/ijms23169022 (PMC9409164; doi:10.3390/ijms23169022)
Supplement: Supplementary file 1 [file ijms-23-09022-s001.zip › ijms-1768729-supplementary.pdf]

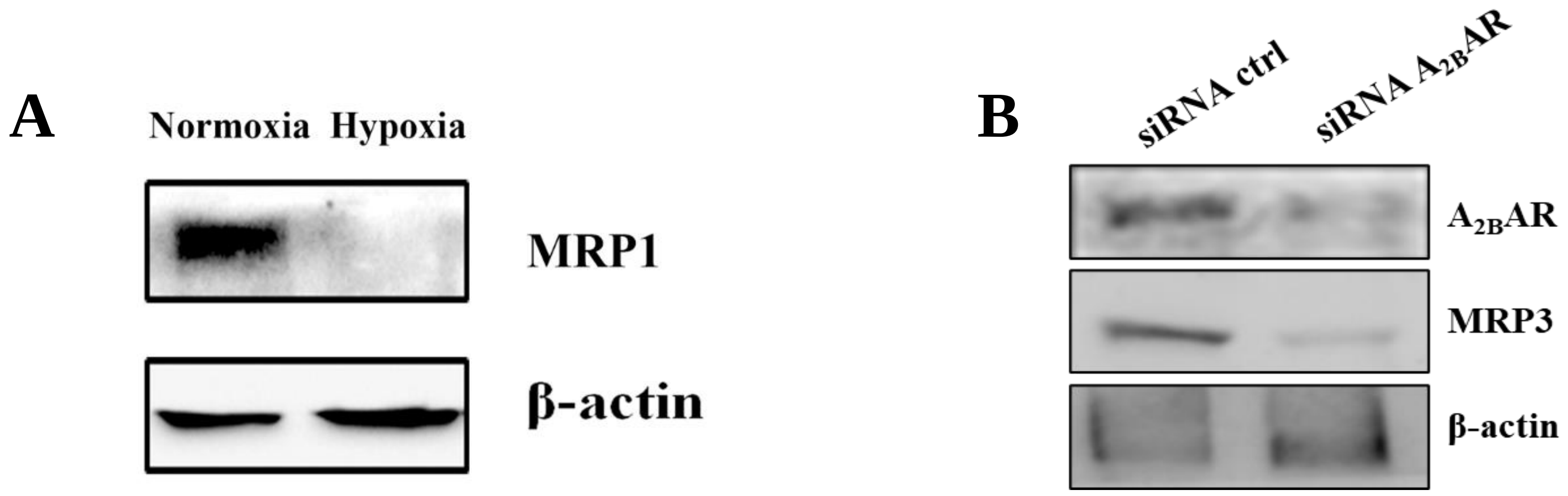

Figure suplementary S1: A) Mrp1 protein expresión in GSCs under normoxia and hypoxia conditions. B) A_{2B}AR and MRP3 knockdown was confirmed by western blot.

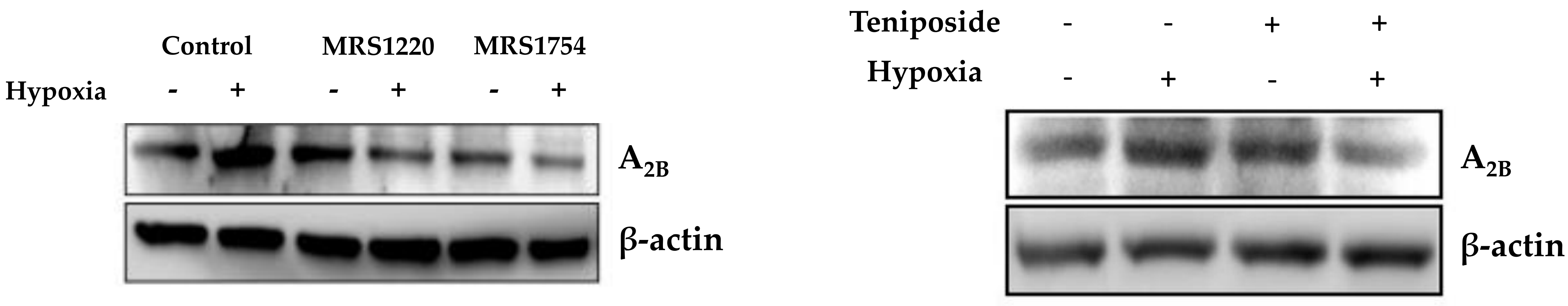

Figure suplementary S2: A_{2B}AR and MRP3 knockdown was confirmed by western blot.
